# Cavernomatous Transformation of the Portal Vein in a Seven-Year-Old Patient: A Case Report

**DOI:** 10.7759/cureus.79390

**Published:** 2025-02-20

**Authors:** Santiago A Mier-Amaya, Luis J Mier-Amaya, Irene García Hernández

**Affiliations:** 1 Transplant and Donation Department, Mexican Social Security Institute, Queretaro, MEX

**Keywords:** acute variceal bleeding, cavernomatous transformation of the portal vein, digestive tract bleeding, non-cirrhotic portal hypertension, portal cavernoma, portal hypertension, portal vein thrombosis, umbilical catheterization

## Abstract

Portal hypertension is a significant cause of upper digestive tract bleeding in pediatric patients. In Mexico, the primary cause of portal hypertension is the cavernomatous transformation of the portal vein (CTPV) secondary to chronic portal vein thrombosis. The portal cavernoma is a morphological anomaly of the portal circulation characterized by dilated and tortuous veins surrounding the porta hepatis formed to circumvent a chronic obstruction. We present the case of a seven-year-old patient with a history of several hospitalizations due to acute upper digestive tract bleeding with subsequent diagnosis confirming the presence of the CTPV. This case underscores the relevance of the pediatric patient history in diagnosing acute upper digestive tract bleeding, emphasizing the need for heightened clinical suspicion and prompt diagnostic and therapeutic intervention in pediatric patients with these characteristics.

## Introduction

The portal vein provides 75% of the liver's total blood flow (800-1200 mL per minute) and is formed by the fusion of the splenic and superior mesenteric veins [1]. Portal hypertension is defined as a pathological increase in pressure of the portal system above 5 mmHg, although it becomes clinically significant when it exceeds 10 mmHg [2].

Portal hypertension can be classified based on its pathophysiological substrate into cirrhotic and non-cirrhotic, with the latter being the most common form in pediatric patients. Non-cirrhotic portal hypertension can be further classified into prehepatic, hepatic, and posthepatic, depending on the subjacent etiology and the location within the portal circulation [2].

In Mexico, the most common form of non-cirrhotic portal hypertension is prehepatic (50%-60%), and it is mainly caused by the cavernomatous transformation of the portal vein (CTPV), also known as portal cavernoma [2]. Moreover, 73% of gastrointestinal bleeding in pediatric patients is of upper gastrointestinal origin, with 6.5%-25% requiring pediatric ICU admission. Only 10% of cases are due to systemic causes, with the remaining cases attributed to local lesions in the digestive tract, such as esophagitis, gastritis, or esophageal varices [3].

Portal cavernoma results from chronic portal vein thrombosis, which obstructs normal blood flow and leads to the development of collateral vessels around the obstruction. These collateral vessels can be identified by Doppler ultrasound as a mass of dilated and tortuous veins surrounding the porta hepatis, specifically in the choledochal venous plexus [4-6].

Portal hypertension in pediatric patients is primarily suspected due to the presence of variceal upper digestive tract bleeding [7,8].

This case report describes a seven-year-old male patient who presented to the emergency department with acute upper gastrointestinal bleeding. The objective is to emphasize the importance of elaborating a complete and thorough medical history in establishing the diagnosis of portal hypertension in children, particularly in regions where complex paraclinical diagnostic procedures are not readily available.

## Case presentation

A seven-year-old male patient presented to the emergency department in January 2025 with diffuse abdominal pain and coffee-ground vomit on three occasions, suggesting acute upper gastrointestinal bleeding.

According to the mother, the patient initially developed clear rhinorrhea, coughing fits, and general malaise on January 1. She consulted a primary care physician who prescribed symptomatic treatment with antipyretics and fluids as needed, but there was no improvement. On January 2, the patient developed acute diffuse abdominal pain, along with three episodes of coffee-ground vomiting, worsening malaise, asthenia, adynamia, and marked pallor, which is why the mother decided to take the patient to the emergency department. Upon physical examination, the patient appeared asthenic, adynamic, and markedly pale. He was hypotensive with a blood pressure of 90/45 mmHg (normal range 100-120/60-75 mmHg) and tachycardic with a heart rate of 140 beats per minute (bpm) (normal range 60-100 bpm). The patient also had a fever, with a temperature of 38.3 °C. He was conscious, cooperative, and showed no signs of respiratory distress, although he had productive coughing fits and bilateral basal crepitus. Hemodynamically, the patient was hypotensive and tachycardic, which was interpreted as hypovolemic shock due to acute upper gastrointestinal bleeding. Abdominal examination revealed diffuse abdominal pain and tenderness, with no signs of peritonitis.

The neonatal history revealed that the patient was born prematurely at 30 weeks of gestation, weighing 1000 g, due to a pregnancy complicated by oligohydramnios and fetal distress, suspected based on a non-reassuring cardiotocography report. He was hospitalized in the neonatal ICU for one month, where he needed mechanical ventilation and umbilical catheterization for 15 days. During his stay, he experienced two cardiorespiratory arrests, the causes of which were not documented in the patient's records.

**Figure 1:**
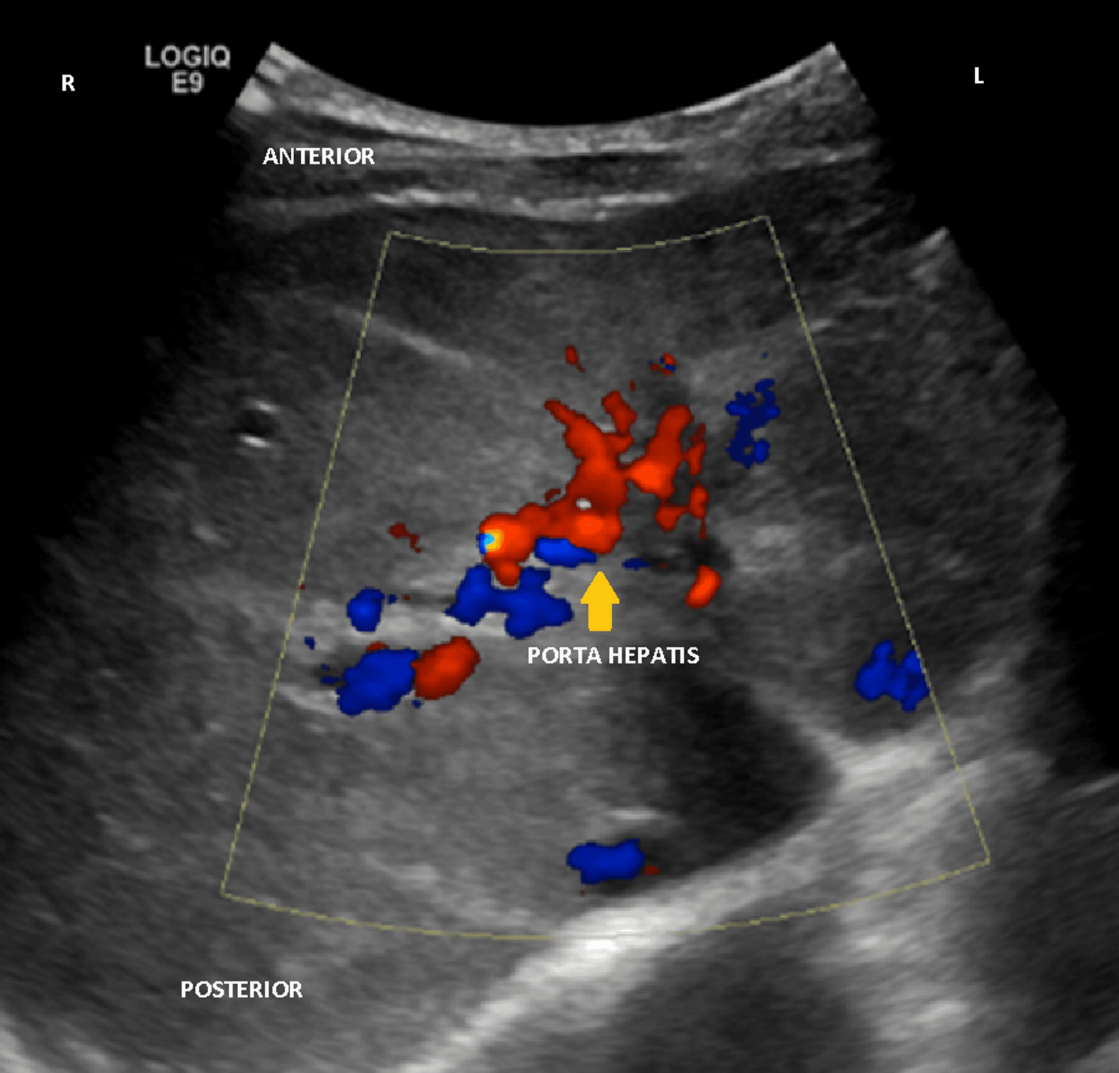
Liver color Doppler ultrasound (July 2022) Doppler ultrasound images showing the dilated and tortuous veins surrounding the porta hepatis (yellow arrow), as well as the distorted anatomy of the portal vein, suggestive of the presence of a portal cavernoma.

Upon further review of the patient's medical records, it was noted that he had been previously hospitalized on four different occasions due to acute upper gastrointestinal bleeding. In March 2022, the patient presented with an acute upper respiratory tract infection, followed by diffuse abdominal pain and melena. During this hospitalization, a hepatic ultrasound revealed chronic diffuse liver disease, as well as an augmented flow in the portal vein that was noted to be surrounded by collaterals. Splenomegaly was also noted, with a spleen diameter of 11.6 cm. The patient was treated with spironolactone, propranolol, and furosemide, showing an appropriate response. An endoscopy performed at that time identified three large esophageal varices with an imminent risk for bleeding, which were ligated. In July 2022, an abdominal ultrasound (Figure 1) showed a liver with a diffuse micronodular pattern and tortuous and dilated collaterals at the porta hepatis suggestive of CTPV, with a sluggish blood flow of 16.1 cm/second (normal range 15-30 cm/second). In November 2022, the patient was hospitalized again after presenting with coffee-ground vomit on several occasions following an upper respiratory tract prodrome. An ultrasound was also performed, revealing the same findings as previously mentioned. In January 2023, the patient was hospitalized for hematemesis and coffee-ground vomiting. He underwent an endoscopy at a local hospital, which identified three esophageal varices occupying 40% of the esophageal lumen. These varices did not disappear with maximal insufflation, and white stelar scars with neoformation vessels were also observed. He was then referred to a concentration center, where another endoscopy was performed, revealing an esophagus with pale pink mucosa and esophageal varices classified as Dagradi III-IV, which were once again transendoscopically ligated. Chronic pangastritis with an erosive and microhemorrhagic component was also noted. The patient began follow-up care with the gastroenterology department and was managed with spironolactone, furosemide, and propranolol. In June 2023, the patient was hospitalized again due to acute upper gastrointestinal bleeding following a prodrome of an acute respiratory tract infection. He underwent another endoscopy to ligate the previously identified esophageal varices.

Laboratory findings were unremarkable, except for the complete blood count that showed anemia with a hemoglobin of 6.2 g/dL (normal range 11.5-13.5 g/dL) and a hematocrit of 19.9% (normal range 35%-40%).

The patient was admitted to the pediatric ICU with the following diagnoses: hemorrhagic shock due to acute upper gastrointestinal bleeding from a variceal origin, portal hypertension, cavernomatous degeneration of the portal vein, and community-acquired pneumonia. He was treated with crystalloid fluid therapy, one unit of packed red blood cells (250 mL), and aminergic support with norepinephrine for the hemorrhagic shock. The patient was placed on *nil per os* and started on an octreotide intravenous infusion for the gastrointestinal bleed, which was further managed with spironolactone, furosemide, and propranolol. His fever was controlled with physical means and acetaminophen. When the patient was stabilized, he was referred to the third-level hospital, where he received gastroenterology follow-up for further definitive treatment.

## Discussion

Umbilical catheterization is the primary method of central venous access in neonatal ICU patients. It is essential for the administration of medication, parenteral nutrition, and blood sample collection. These catheters can be placed up to five days after birth and may be used for up to 14 days, although they should be removed as soon as possible to minimize the risk of complications [9,10].

Noninfectious complications include arrhythmias (mainly atrial flutter and supraventricular tachyarrhythmias), cardiac tamponade, pulmonary embolism, and various portal circulation disorders, particularly portal pneumatosis and portal vein thrombosis. Approximately 30% of patients with portal vein thrombosis have a history of umbilical catheterization [10]. Portal vein thrombosis due to umbilical vein catheterization can result from direct mechanical damage to endothelial cells during catheter placement or chemical damage caused by the infusion of hyperosmolar substances [9,10]. The patient described in this case report underwent 15 days of umbilical catheterization, which, as previously mentioned, exceeds the recommended duration for safe use.

Portal vein thrombosis is the primary cause of prehepatic portal hypertension in pediatric patients [2]. The most common manifestations in patients with portal hypertension include digestive tract bleeding, hematemesis, melena, and splenomegaly, typically presenting around the age of 4.6 years [6]. This is consistent with our patient, who experienced his first gastrointestinal bleeding at the age of 4 years and 10 months. These manifestations occur due to the splanchnic blood flow redistribution through systemic collaterals, which promotes the development of varices that are prone to bleeding [7,8]. Patients with variceal bleeding tend to present after an episode of febrile disease, such as an upper respiratory tract infection. It is believed to be due to the use of nonsteroidal anti-inflammatories for symptomatic control and the increase in splanchnic pressure due to cough paroxysms [8].

The primary actions for managing a patient with acute digestive tract bleeding are to stabilize the patient, secure the airway, and initiate fluid resuscitation to maintain adequate blood pressure. Transfusion of packed red blood cells is recommended to achieve a hemoglobin level of 7-9 g/dL to avoid overtreatment, as excessive transfusion may increase portal pressure and worsen the bleeding. Fresh frozen plasma, platelets, and vitamin K can be used to correct coagulopathies, if present. Pharmacologically, somatostatin analogs such as octreotide are recommended to reduce splanchnic blood pressure and control bleeding. Finally, once the patient is stable, an endoscopy should be performed to identify and treat the bleeding source, either through sclerotherapy or band ligation [7,8].

The case presents a pediatric patient with upper digestive tract bleeding of variceal origin. Diagnostic workup revealed that the patient had portal hypertension caused by chronic portal vein thrombosis, as confirmed by Doppler ultrasound during the patient's first hospitalization. Notably, each bleeding episode was preceded by an acute upper respiratory tract infection, consistent with the reviewed evidence, probably due to the coughing fits and the use of nonsteroidal anti-inflammatory drugs. In all four events, the patient was stabilized using the standardized treatments described in the literature and had a favorable outcome.

The underlying cause of the patient's chronic portal vein thrombosis can be traced back to his neonatal history, where it is shown that the patient was subject to intensive care, which required the placement and prolonged use of an umbilical vein catheter, from which thrombosis is a well-known and documented complication.

## Conclusions

Acute digestive tract bleeding is a dramatic complication of portal hypertension. While well known and studied in adults, its presentation in pediatric patients can be shocking, particularly for primary care physicians. We present the case of a seven-year-old male patient with a history of several bleeding events, along with a brief summary of the diagnostic approach used to identify the source of bleeding. A rapid review of the literature on portal hypertension and CTPV is also included.

The presence of coffee-ground vomit, hematemesis, and/or melena should prompt the physician to strongly suspect acute upper gastrointestinal bleeding. The most important intervention in a primary-care setting is immediate referral to the emergency department, where the patient can be stabilized and further evaluated to identify the source of bleeding. In pediatric patients, the presence of esophageal varices should immediately suggest portal hypertension. Portal hypertension can be caused by various pathologic entities, which can be classified as prehepatic, intrahepatic, and posthepatic, depending on their location in the portal circulation. The primary cause of prehepatic portal hypertension is portal cavernoma. Further research is needed to establish institutional safety protocols regarding the use of umbilical catheterization, as well as to create a standardized approach for patients with CTPV. This would allow for the development of clear, straightforward algorithms to guide diagnostic and therapeutic interventions.
